# The Role of Integrins in Cancer and the Development of Anti-Integrin Therapeutic Agents for Cancer Therapy

**Published:** 2008-04-10

**Authors:** Xinjie Lu, Dong Lu, Mike Scully, Vijay Kakkar

**Affiliations:** 1 Thrombosis Research Institute, Manresa Road, London, SW3 6LR U.K; 2 The Wellcome Trust Sanger Institute, Wellcome Trust Genome Campus, Hinxton, Cambridge, CB10 1SA, U.K

**Keywords:** integrin, cancer, disintegrin, ligand

## Abstract

Integrins have been reported to mediate cell survival, proliferation, differentiation, and migration programs. For this reason, the past few years have seen an increased interest in the implications of integrin receptors in cancer biology and tumor cell aggression. This review considers the potential role of integrins in cancer and also addresses why integrins are present attractive targets for drug design. It discusses of the several properties of the integrin-based chemotherapeutic agents currently under consideration clinically and provides an insight into cancer drug development using integrin as a target.

## Introduction

Integrins are a large family of eukayotic cell-surface receptors that mediate dynamic interaction between cells and extracellular adhesion molecules (Humphries, 2000). The integrins recognize extracellular matrix (ECM) proteins or counter-receptors on adjacent cells. ECM molecules that affect cell adhesion include glycoproteins such as fibronectin (Fn) (Gardner and Hynes, 1985), von Willebrand factor (vWF) (Chow et al. 1992), vitronectin (Vn) (Pytela et al. 1985), thrombospondin (Tsp) (Karczewski et al. 1989), tenascin (Tn) (Joshi et al. 1993), collagen (Coll) (Heino, 2000), laminin (Ln) (Burgeson and Christiano, 1997), osteopontin (Opn) (Green, 2001), and other unidentified molecules. A key finding in the discovery of the integrins was that the well known amino acid sequence Arg-Gly-Asp (RGD) which was initially found in fibronectin, serves as a primary cell recognition motif. Subsequently, the RGD sequence was found in many ECM molecules and, in many cases, was responsible for cell attachment (Karczewski et al. 1989; Joshi et al. 1993; Ruggeri et al. 1983; Davis, 1992; Schnapp et al. 1995; Kimura et al. 1998). The recent crystal structures of the extracellular domains of α_V_β_3_ (Xiong et al. 2001; 2002) have provided new insights into integrin activation and ligand recognition. The interaction of integrins with their ligands is dependent upon signals transduced from the cytoplasmic tails to the extracellular domains (Travis et al. 2003). The binding of integrins to their ligands is critically important to many diverse physiological phenomena, such as attachment, cell proliferation (Miyata et al. 2000; Hollenbeck et al. 2004; Hedin et al. 2004; Zhou et al. 2004), migration (Hirsch et al. 1996; Sakai et al. 1998; Fujiwara et al. 2001; Paulhe et al. 2001). Integrins also contribute to the initiation and/or progression of many diseases including tumor invasion, angiogenesis and metastasis (Tsuji et al. 2004; Takanami et al. 2005; Guo et al. 2005; Enserink et al. 2004; Chung et al. 2004; Felding-Habermann et al. 2002; Gladson et al. 1996; Zheng et al. 1999; Zheng et al. 2000).

## Integrin Family

Integrin family have atleast 18 α- and 8 β-subunits that are known to comprise least 24 members (Berman et al. 2000; Parise et al. 2000; Humphries, 2000; Hynes, 2002). Additionally, in a recent survey of human genome, 24 α- and 9 β-subunits have been identified (Venter et al. 2001), which implies 6 novel α- and 1 novel β-subunits. However, their existence is not yet firmly established. Integrins are found in many species, ranging from sponges to mammals (Brower et al. 1997; Gettner et al. 1995).

Cell adhesion requires integrin occupancy. The binding of integrins to their ligands has been intensively studied employing proteolytic fragments and synthetic peptides corresponding to selected regions in Fg, Fn and several other matrix components (Table 1).

As shown in Table 1, the two distinct subunits form noncovalent heterodimers where each subunit has a large extracellular domain (700–100 residues), a single transmembrane domain and a short cytoplasmic domain (20–70 residues). The exception to this is the β_4_ subunit which has an extended cytoplasmic domain containing four Fn type III-like domains (Colombatti et al. 1993). All integrin dimers are dissociated by ionic detergents, indicating that the subunits are noncovalently held together.

The α subunits are subdivided into two groups based on some structural differences. The first group is comprised of the α_1_, α_2_, α_D_, α_E_, α_L_, α_M_ or α_X_ subunits, respectively. The second group is composed of α_3_, α_4_, α_5_, α_6_, α_7_, α_8_, α_9_, α_10_, α_11_, α_IIb_ or α_V_ subunits, respectively, and is bridged by a disulphide bond with an exceptional α_4_ subunit subjected to a post translational cleavage at a site close to the transmembrane domain of the precursor. Thus, there are two chains linked by a disulphide bridge, a light chain and a heavy chain. The light chain is composed of the cytoplasmic domain, the transmembrane region and a part of the extra-cellular domain (about 25 kD), while the heavy chain contains the rest of the extracellular domain (about 120 kD). Integrin α_4_ is unique among all known integrin α subunit sequences in that it (i) has neither an inserted I-domain, nor a disulfide-linked C-terminal fragment, and (ii) a potential protease cleavage site, near the middle of the extra-cellular portion of the polypeptide rather than close to the transmembrane domain of other integrin α subunits (Xiong et al. 2001, 2002).

The β_1_ integrins generally mediate interaction between cells and ECM (Perlino et al. 2000). The β_2_ integrins subfamily including α_L_β_2_, α_M_β_2_, α_X_β_2_, α_D_β_2_, are immunologically restricted to leukocytes and typically have other cell surface molecules as their ligands, for example, α_L_β_2_ interacts with counter receptors ICAM-1, ICAM-2, and ICAM-3 (Marlin and Springer, 1987; de Fouerolles and Springer, 1992; Binnerts et al. 1996; Woska et al. 1996) while α_M_β_2_ recognizes iC3b (Wright and Silverstein, 1983), fibrinogen (Zhang and Plow, 1996) and neutrophil inhibitory factor (NIF) (Muchowski et al. 1994). There are two integrins α_IIb_β_3_ and α_V_β_3_ in integrin family, that share the common β_3_ subunit, have been reported to function as promiscuous receptors for the RGD-containing adhesive proteins such as fibrinogen, vitronectin, fibronectin, von Willebrand factor, and thrombospondin (Scarborough et al. 1999). The β_4_ integrin facilitates key functions of carcinoma cells, including their ability to migrate, invade, and evade apoptosis (Folgiero et al. 2007). The β_5,_ β_6,_ β_7,_ β_8_ and β_N_ subunits can form a dimmer with an α_V_ subunit binding to different ligands (Table 1) and showing different functions.

## Integrin Structure

**Figure 1:**
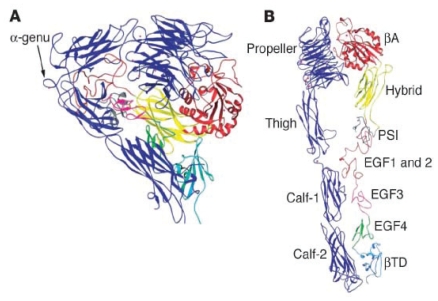
Structure of the extracellular segment of α_V_β_3_ derived with permission from Xiong et al. 2002. (**A**) Bent conformation of α_V_β_3_ as it was present in the crystal. (**B**) Extension of the structure to reveal its domains.

The first three-dimensional structure of the extracellular domain of an integrin was published in October 2001, a decade and a half after the family was first defined (Xiong et al. 2001, 2002, 2004) (Fig. 1).

Crystal structure of integrin α_V_β_3_ showing the dimer and individual subunits (Xiong et al. 2002). An unliganded ectodomain from the αA-lacking integrin α_V_β_3_ contains the two subunts assembled into a globular head built by two predicted domains: the N-terminal seven-bladed propeller domain of α_V_ and an αA-like domain (βA) from the β_3_. βA loops out from the “Hybrid” domain (β_3_ residues 55–108 and 353–432), which itself is inserted in the N-terminal plexin/semaphorin/integrin (PSI) domain (residues 1–54 and residues 433–435) of β_3_. The PSI domain and the beta-tail domain (βTD), together forming the β_3_ leg. Ig-like thigh domain and calf-1 and calf-2 domains formed the α_V_ leg. Two legs are bent at the “knees” and folded back against the head of the same molecule. This sharp bending takes place between the thigh and calf-1 of α_V_ (α-genu) and approximately corresponding to between EGF domains 1 and 2 of β_3_ (β-genu). A metal ion (Ca^2+^ or Mn^2+^) occupies the α-genu on both the ligand and unliganded structures. At the base of propeller, blades 4–7 each contain a metal ion coordinated in a β-hairpin loop.

## Integrin and Cancer

Cancer occurs when cells become abnormal and keep dividing and forming more cells without control or order. If cells keep dividing when new cells are not needed, a mass undifferentiated tissue forms. This mass of extra tissue, called a growth or tumour, can constitute either a benign or a malignant tumour respectively. Benign tumors can usually be removed and, in most cases, they do not come back. Most importantly, cells from benign tumors do not spread to other parts of the body. Benign tumors are rarely a threat to life. In contrast, malignant tumours are truly cancerous. Cancer cells can invade and damage nearby tissues and organs. Cancer cells can break away from a malignant tumor and enter the bloodstream or the lymphatic system. The spread of cancer is called metastasis which appears to be a complex multistep process that involves the invasion of cancer cells from primary neoplasm followed by their dissemination through the lymphatic vessels and systemic circulation. New blood vessels form either by vasculogenesis, which refers to initial events of vascular growth in which endothelial cell precursors (angioblasts) differentiate and assemble into primitive vessels or by angiogenesis, which refers to a combination of sprouting of new vessels from pre-existing ones, and longitudinal separation of pre-existing vessels in a process named intussusception (Conway et al. 1993). The angiogenesis can be triggered in pathological conditions such as tumor growth and chronic wounding. Angiogenetic process involves functional cooperativity between cytokines and endothelial cell (EC) surface integrins. Cell bound integrins by their physical interaction with ligands necessary are essential for cell adhesion, migration and positioning, and induce signaling events essential for cell survival, proliferation and differentiation. They also trigger a variety of signal transduction pathways which are involved in mediating invasion, metastasis and squamous-cell carcinoma which can be reviewed as follows. The review focuses mainly on specific α and β subtypes which have been most extensively investigated in cancer.

### β_1_ class of integrins

Although little clear correlation between tumor formation, invasion and β_1_ integrin expression has yet been demonstrated in human patients, it has been possible to show a crucial role of β_1_ integrin in tumor formation and metastasis in mice. Tumor cells expressing β_1_ integrin formed significantly larger primary tumors and had a dramatically increased metastasis into liver and lung (Brakebusch et al. 1999). In another study, which used a T cell lymphoma line in which both β_1_ integrin alleles was deleted by homologous recombination, metastasis formation in mice was significantly reduced (Stroeken et al. 2000). Recently, it was shown that ablation of the β_1_ integrin gene in mammary epithelium dramatically impaired mammary tumorigenesis in mice (White et al. 2004). Sudhakar et al. have reported that human collagen α1(IV)NC1 binds to α_1_β_1_ integrin, competes with type IV collagen binding to α_1_β_1_ integrin, and inhibits migration, proliferation, and tube formation by ECs, indicating that α1 (IV)NC1 is a potential therapeutic candidate for targeting tumor angiogenesis (Sudhakar et al. 2005). A study using β_1_ integrin double knockout lymphocytes and retransfection of β_1_ integrin deletion mutants have shown that different parts of the cytoplasmic domain of β_1_ integrin are required either for adhesion or for invasion and metastasis (Stroeken et al. 2000).

Integrin α_1_β_1_ and α_2_β_1_ were shown to regulate hepatocarcinoma cell invasion across the fibrotic matrix microenvironment (Yang et al. 2003). A potent selective inhibitor of α_1_β_1_ integrin, obtustatin purified from the venom of the *Vipera lebetina obtusa* viper was reported to have a marked ability to inhibit angiogenesis in vivo in the chicken chorioallantoic membrane assay, and in the Lewis lung syngeneic mouse model (Marcinkiewicz et al. 2003). Grzesiak and Bouvet have demonstrated that the certain cancer cell lines including CFPAC (a ductal epithelioid cell line established from a cystic fibrosis patient with pancreatic adenocarcinoma), BxPC-3 (human pancreas adenocarcinoma), Colo-357 (human lymph node metastasis), and Panc-1 (Pancreatic Cancer Cell Line) attach to 3D type I collagen scaffolds in an α_2_β_1_-specific manner and that this integrin-specific adhesion is required for subsequent cell proliferation. Such evidences support the notion that targeting α_2_β_1_ integrin-specific type I collagen adhesion may have therapeutic value in the treatment of pancreatic cancer (Grzesiak and Bouvet, 2007). Integrin α_2_β_1_ was also reported to mediate the anti-angiogenic and anti-tumor activities of angiocidin, a novel tumour-associated protein which is capable of binding to both α_2_β_1_ and type I collagen. This protein promoted α_2_β_1_-dependent cell adhesion and inhibited tumor growth and angiogenesis (Sabherwal et al. 2006). Combined antagonism of α_1_β_1_ and α_2_β_1_ was shown to reduce tumor growth substantially as well as angiogenesis of human squamous cell carcinoma xenografts (Senger et al. 2002).

The interaction of α_3_β_1_ with ligand laminin-5 has been demonstrated to promote the migration and invasion of malignant glioma and melanoma cells (Tsuji, 2004; Tsuji et al. 2002; Giannelli et al. 2007) and to promote binding to virus glycoprotein. A significant increase in proliferation and adhesion in response to collagen 1 and laminin for integrin receptor α_3_β_1_ was also observed in ovarian cancer cell lines (Ahmed et al. 2005). More recently, uPAR (urokinase-type plasminogen activator receptor), and TIMP (tissue inhibitors of metalloproteinases)-2 were also proposed as ligands of α_3_β_1_ integrin in mediating uPA/uPAR interaction and intracellular signaling (Wei et al. 2007). In an animal model it was shown that soluble uPAR antagonizes cancer progression (Jo et al. 2003).

The Src family kinases are classified as oncogenic proteins due to their ability to activate cell migration (Rodier et al. 1995; Rahimi et al. 1998) in many cell types including epithelial tumor cells. Studies with chimeric α_4_ integrin subunits have shown that α_4_ cytoplasmic domain can enhances cell migration via c-Src activation (Chan et al. 1992; Hsia et al. 2005).

α_5_β_1_ integrin interacts with Fn which is implicated in several cellular activities including cell proliferation, differentiation, and migration. A high-affinity interaction that occurs with the central cell binding domain, a region involved in many fundamental aspects of cell growth and morphogenesis, is dependent on the RGD sequence and other recognition sequences (Li et al. 2003; Murillo et al. 2004). The interaction with Fn has been demonstrated with both lung epithelial cells and fibroblasts. In addition, the inhibition of cell surface α_5_ integrin expression was found to decrease phosphoinositide-3 kinase (PI3K) activity and inhibit colon cancer cell attachment, suggesting that agents which selectively target α_5_ integrin subunit expression may enhance the effects of standard chemotherapeutic agents and provide a novel adjuvant treatment for selected colon cancers (Lopez-Conejo et al. 2002). Furthermore, cells expressing the α_5_β_1_ integrin displayed a dramatic enhancement in the ability of growth factors to activate PI3K and protein kinase B (PKB), indicating this stimulation may also involve the interaction between α_5_β_1_ and the PI3 K and PKB signalling pathways (Lee et al. 2000). Wei et al. recently reported that urokinase receptor binding to α_5_β_1_ is required for maximal responses to Fn and tumor cell invasion (Wei et al. 2007). Kuwada et al. demonstrated that expression of integrin α5β1 in colon cancer cells decreases HER (human epidermal growth factor receptor)-2-mediated proliferation, crystal violet assays were showing inhibition of the cell proliferation of Caco-2 control cells with the antagonistic HER-2 antibody mAb 4D5 (Kuwada et al. 2005). MAb 4D5 is also indicated clinically active in cancer patients to target HER2-overexpression (Baselga et al. 1996; Rhodes, 2005). Furthermore, mAb 4D5 has been shown great promise as targeted agents in the treatment of patients with cancer (Bartsch et al. 2007).

It has been reported that α_6_ integrin-mediated neutrophil migration through the perivascular basement membrane (PBM) is platelet-endothelial cell adhesion molecule1 (PECAM-1) dependent, a response associated with PECAM-1-mediated increased expression of α_6_β_1_ on transmigrating neutrophils (Dangerfield et al. 2002). Significantly increased ovarian cancer cell line proliferation and adhesion to collagen 1 and laminin (ligands of integrin receptor α_6_β_1_) were also reported (Ahmed et al. 2005). In addition, an α_6_ integrin is found to be overexpressed in human oesophageal carcinomas, suggesting an important role in oesophageal tumor invasion (Tanaka et al. 2000). This notion has since been confirmed by other studies (Mercurio et al. 2001; Demetriou et al. 2004).

The α_7_β_1_ integrin is a laminin-binding receptor that was originally identified in melanoma (Kramer et al. 1991). Ziober et al. reported that during melanoma progression, acquisition of a highly tumorigenic and metastatic melanoma phenotype is associated with loss of the α_7_β_1_ (Ziober et al. 1999). Integrin α_7_β_1_ serves an important mechanical function in the diaphragm by contributing to passive compliance, viscoelasticity, and modulation of muscle contractile properties (Lopez et al. 2005).

Integrin α_10_β_1_ is a major collagen-binding integrin during cartilage development and in mature hyaline cartilage while α_11_β_1_ was originally found in fetal muscle (Gullberg et al. 1995). Integrin α_11_β_1_ recognizes the triple-helical GFOGER sequence (where single letter amino acid nomenclature is used, O = hydroxyproline) found in interstitial collagens (Tulla et al. 2001). Little is known about the biology of these recently identified integrins. Integrin α_10_β_1_ is expressed on chondrocytes and some fibrous tissues. Integrin α_11_β_1_ is involved in cell migration and collagen reorganization in mesenchymal non-muscle cells (Tiger et al. 2001). Recently, α_11_β_1_ integrin is required on periodontal ligament fibroblasts for cell migration and collagen reorganization by assisting axial tooth movement (Popova et al. 2007).

### α_V_ class of integrin

The first integrin found associated with tumor angiogenesis was α_V_β_3_ (Eliceiri et al. 1998; Eliceiri, 2001; Ruegg et al. 2003). Integrin α_V_β_3_ has a broad distribution and is found on endothelial cells, smooth muscle cells (SMCs) and hematopoietic cell types such as platelets and osteoclasts. The interaction of α_V_β_3_ with its ligands plays a crucial role in angiogenesis and neointimal formation after vascular injury. In addition, during osteoclast-mediated bone resorption, α_V_β_3_ regulates the cytoskeletal organization required for cell migration and formation of the sealing zone (McHugh et al. 2000). Prostate cancer specific integrin α_V_β_3_ was demonstrated to modulate bone metastatic growth and tissue remodeling (McCabe et al. 2007). The study of co-expression of bone sialoprotein, integrin α_V_β_3_, and MMP-2 in papillary thyroid carcinoma cells demonstrated that cancer cells appear to become more invasive when bone sialoprotein forms a cell-surface trimolecular complex that links MMP-2 to integrin α_V_β_3_ (Karadag et al. 2004). Bayless et al. present very convincing data showing that integrin α_V_β_3_ as well as integrin α_5_β_1_ regulate human endothelial cell vacuolation and lumen formation, implicating a major role contributed by these two integrins for endothelial cell morphogenesis (Bayless et al. 2000). It is also clear that the integrin α_V_β_3_ plays an important role in virtually every stage of cancer progression. Indeed, neuroblastoma aggressiveness has been identified to be correlated with the expression of integrin α_V_β_3_ and α_V_β_5_ by microvascular endothelium (Erdreich-Epstein et al. 2000). Other studies also demonstrate that increased α_V_β_3_ expression level is closely associated with increased cell invasion and metastasis (Feldin-Habermann et al. 2002). Li et al. reported that antisense α_V_ suppressed tumour growth more strongly than antisense β_3_, antisense therapy but simultaneous targeting at both integrin subunits was more effective than the respective monotherapies (Li et al. 2007). Integrin α_V_β_3_ has been demonstrated to interact with the activated forms of the platelet-derived growth factor, insulin, and vascular endothelial growth factor (VEGF) cell receptors faciliting optimal activation of cell proliferative signalling pathways (Giancotti and Ruoslahti, 1999; Kumar, 2003). The functional activity of αvβ3 on endothelial and tumor cells may well be regulated by VEGF (Byzova et al. 2000). VEGF has been also implicated in prostate carcinogenesis and metastasis as well as in angio-genesis. Both VEGF and its receptor are expressed by prostate carcinoma cells at a high level (Ferrer et al. 1998; 1999).

A role for α_V_β_6_-mediated production in the regulation of MMP-9 and MMP-3 have been reported in several tumor types and in untransformed keratinocytes (Ramos et al. 2002; Ahmed et al. 2002). MMP-9 plays a critical role in the recruitment of bone marrow derived CD45 positive cells into the primary tumor and the establishment of a mature vasculature (Jodele et al. 2005). Integrin α_V_β_6_ also plays a role in wound healing and cancer of the oral cavity (Thomas et al. 2006). In addition, α_V_β_6_ has been implicated in the regulatory control of the uPA proteolytic cascade (Ahmed et al. 2002). A gradual increase in the expression of α_V_β_6_ integrin from borderline to malignant tumors has been reported in oral squamous carcinomas (Jones et al. 1997) and breast carcinomas (Arihiro et al. 2000). In malignant keratinocytes and colon cancer cells, increased expression of this integrin enhances MMP-9 secretion and MMP-9-mediated invasion (Thomas et al. 2000; Agrez et al. 1999). Inhibition of α_V_β_6_ function using inhibitory antibodies results in total abrogation of MMP-9 activation (Thomas et al. 2001) suggesting that the expressions of α_V_β_6_ integrin and MMP-9 are linked, and their coordinate expression appears to promote invasion by squamous and colon carcinoma cells. The integrin α_V_β_6_ interacts with Fn, Vn (Huang et al. 1998), tenascin (Weinacker et al. 1995), and latency-associated peptide (Munger et al. 1999), a protein derived from the N-terminal region of the transforming growth factor(TGF)-β gene product that mediates cell adhesion, spreading, migration, proliferation, and activation of latent TGF-β (Weinreb et al. 2004; Thomas et al. 2001).

Until recently, there has been little information about integrin α_V_β_8_ which has been reported to function as an additional receptor for foot-and-mouth disease virus (FMDV) (Jackson et al. 2004) in addition to the three RGD-dependent integrins α_V_β_1_, α_V_β_3_, and α_V_β_6_, which have been shown to be receptors for FMDV previously (Jackson et al. 1997, 2000, 2002; Duque et al. 2004). Notably, α_V_β_8_ as well as α_V_β_6_ may promote epithelial-mesenchymal transition (EMT) by contributing to the activation of TGF-β (Munger et al. 1999). Additionally, α_V_β_8_—mediated activation of TGF-β was shown to block the proliferation of certain cancer cells (Mu et al. 2002). Several recent studies have demonstrated that both up-regulation and down-regulation of expression of α_V_ integrins and other integrins can be effective markers of malignant diseases and patient prognosis.

Although there are few reports of enhanced expression of α_IIb_β_3_ (than of α_V_β_3)_ integrin in tumour cells, one observation indicated an important role in tumour progression. A study on human melanoma biopsies showed that α_IIb_β_3_ expression increased with tumour thickness (Trikha et al. 2002a). In addition, a single pretreatment of human melanoma cells with c7E3 Fab, an α_IIb_β_3_ antibody inhibited lung colonization of the tumor cells in severe combined immunodeficient mice (Trikha et al. 2002b).

### Other sub-classes of integrins

Parathyroid hormone-related protein (PTHrP) was reported to not only increase transcriptional activity of the integrin subunit α_5_ (Anderson et al. 2007) but also upregulate integrin α_6_β_4_ expression and activate Akt in breast cancer cells (Dittmer et al. 2006; Shen and Falzon, 2006; Shen et al. 2007). Falcioni et al. first identified a tumor antigen (TSP-180) associated with metastasis that was shown to be identical to the β_4_ integrin subunit (Falcioni et al. 1997; Kennel et al. 1989). Subsequently other studies showed that expression of α_6_β_4_ persists in some aggressive carcinomas and that its expression may be linked to the behavior of these tumors (Guo et al. 2004). At earlier of the year in 2001, Davis and his colleagues demonstrated that α_6_β_4_ integrin has an influence on tumour biology as this integrin and its ligand, laminin-5, are essential gene products for the maintenance and remodeling of a stratified epithelium (Davis et al. 2001). The β_4_ integrin, for example, was lost in the lesions of prostatic intraepithelial neoplasia together with basal cell-lining and in prostate carcinoma the expression of β_4_ integrins was totally lost (Davis et al. 2001). In normal skin keratinocytes, expression of the α_6_β_4_ integrin is restricted to the proliferative basal layer and mediates stable adhesion to the underlying basement membrane. Observations in carcinoma cells show a functional and spatial dissociation of the α_6_β_4_ integrin from the hemidesmosomal complex, which stimulates cell migration and, therefore, may contribute to carcinoma invasion (Kippenberger et al. 2004). Indeed, many carcinomas express elevated levels of α_6_β_4_ (Herold-Mende et al. 2001), particularly breast carcinomas (Chung and Mercurio, 2004).

Pawar et al. recently have shown that the uPA-mediated cell surface cleavage of the alpha6 integrin extracellular domain is involved in tumor cell invasion and migration on laminin (Pawar et al. 2007). In addition, observations in carcinoma cells show a functional and spatial dissociation of the α_6_β_4_ integrin from the hemidesmosomal complex, which stimulates cell migration (Kippenberger et al. 2004). Furthermore, blocking antibodies to either α_6_ or β_4_ integrin subunits suppress the formation of apoptosis-resistant acinar structures in Matrigel by mammary epithelial cells (Weaver et al. 2002), suggesting a role for β_4_-mediated cellular polarity in mediating antiapoptotic signaling. Integrin α_6_β_4_ was recently noted only at the cell’s basal interface with the basement membrane in normal pancreatic ducts. But in pancreatic adenocarcinomas, 92% demonstrated overexpression of integrin α_6_β_4_ and altered localization to the cytoplasm and membranous regions, this upregulation and redistribution of integrin α_6_β_4_ expression implicated a role of integrin in pancreatic adenocarcinoma progression (Cruz-Monserrate et al. 2007). Interestingly, the expression of β_4_ was inversely correlated with dissemination of ten human gastric cancer cell lines in SCID (severe combined immunodeficiency) mice (Ishii et al. 2000). In addition, strong evidence suggests that reduced expression of α_6_ and β_4_ subunits may contribute to the higher tumorigenicity of androgen-independent prostate tumor cells (Bonaccorsi et al. 2000).

Several key signalling molecules in carcinoma cells are also involved in the mechanisms of α_6_β_4_ integrin-mediated tumour behaviour (Bon et al. 2007; Folgiero et al. 2007) since β_4_ has been demonstrated to interact with ERBB2 (erythroblastic leukemia viral oncogene homolog 2, encoding an 185-kDa, 1255 amino acids, orphan receptor tyrosine kinase) that displays potent oncogenic activity when overexpressed) in some cultured breast tumour cells, and the two proteins synergize in promoting cellular proliferation and invasion (Falcioni et al. 1997). In addition, Guo et al. established that integrin α_6_β_4_ may be required for mammary tumourigenesis driven Lu et al by the expression of ErBB2 (Guo et al. 2006). Folgiero et al. revealed that α_6_β_4_ can regulate the expression of ErBB-3 at the level of protein translation, resulting in a significant induction of ErBB-2/ErBB-3 heterodimerization and consequent activation of PI3K (Folgiero et al. 2007; Liu et al. 2007). Introduction of β_4_ in β_4_-negative breast carcinoma cells activates signalling from PI3K to Rac (a member of the Rho family of small guanosine triphosphatases) and increases the invasion of these cells in vitro (Shaw, 2001).

The integrin α_E_β_7_ (also known as cell marker CD103) is expressed by most intra-epithelial lymphocytes (IEL). An important ligand for this molecule is the epithelial cell adhesion molecule E-cadherin. Cresswell et al. have demonstrated that the up-regulation of integrin α_E_β_7_ by lymphocytes increases adhesion to E-cadherin expressing bladder cancer targets, indicating a role of integrin α_E_β_7_ in cancer invasion (Cresswell et al. 2002).

## Integrins as Targets for the Treatment of Cancer

From what has been discussed above, integrins play a key role in tumor angiogenesis and cancer. Because they are cell surface receptors interacting with extracellular ligands, they represent ideal pharmacological targets. A variety of integrin antagonists such as low molecular weight inhibitors, peptidomimetics, or monoclonal antibodies are in various stages of development as anti-cancer therapeutics (Kerr et al. 2002; Mousa 2002; Tucker et al. 2003).

In-vivo study has demonstrated that the addition of inhibitory anti-β_1_-integrin antibodies or the re-expression of α_2_β_1_ integrins leads to the reversal of the malignant phenotype in a 3-dimensional cell culture model and to a reduction in tumour formation in animal models (Zutter et al. 1995). Yao et al. recently show that β_1_ integrin expression has potential prognostic value in invasive breast cancer and that coexpression of fibronectin may help identify patients with more aggressive tumors who may benefit from targeted therapy (Yao et al. 2007). More studies have been focused on α_V_β_3_, since α_V_β_3_ has been identified as a prognostic indicator of survival and a specific potential target for control of angiogenesis, therapies directed against integrin against α_V_β_3_, have been developed (Brooks et al. 1994; Gladson et al. 1996; Zhang et al. 2007; Gramoun et al. 2007).

## Antibodies Against Integrins as Inhibitors

### MEDI-552

Brooks and his coworkers first showed that a monoclonal antibody specific for α_V_β_3_, MEDI-552 (LM609), could block angiogenesis in a murine model (Brooks et al. 1994). In addition, there is an ongoing phase I dose escalation study evaluating the safety of MEDI-522 in patients with advanced malignancies. This antibody was chosen for its unique ability to selectively target multiple and different cell types. In a phase I trial on various solid tumours, MEDI-522 appeared to be without significant toxicity (McNeel et al. 2005). MEDI-522 was detectable both in quiescent and in angiogenically active skin blood vessels as well as in the dermal interstitial space. The levels of phosphorylated focal adhesion kinase (pFAK) were reduced during MEDI-522 treatment, suggesting a modulating effect on this signaling molecule (Zhang et al. 2007; Gramoun et al. 2007).

### CNTO 95

A fully humanized monoclonal antibody to anti-α_V_ integrins, CNTO 95, has been shown to inhibit angiogenesis and tumor growth in preclinical studies (Mullamitha et al. 2007). CNTO 95 is likely to be less immunogenic in humans compared to chimeric or humanized antibodies (Trikha et al. 2004). CNTO 95 bound to purified α_V_β_3_ and α_V_β_5_ with higher affinity (a Kd of approximately 200 pM and to α_V_ integrin-expressing human cells with a Kd of 1–24 nM). In vitro, CNTO 95 potentially inhibited human melanoma cell adhesion, migration and invasion (doses ranging 7–20 nM) and appeared to be safe without inhibition of normal physiologic angiogenesis (Martin et al. 2005; Trikha et al. 2004).

### 17E6

The 17E6 antibody strongly perturbs cell attachment mediated by α_V_ associated integrins, by reacting with α_V_β_3_, α_V_β_5_, and α_V_β_1_, and has the ability to disrupt stable interaction between vitronectin and α_V_β_3_, and blocks the growth of M21 tumours in nude mice. In two nude mouse tumor models, injection of 17E6 strongly inhibited tumor development (Mitjans et al. 2000; Mitjans et al. 1995).

Integrin antibodies that block specific integrins for treatment of cancer are still in clinical trial stages as lessons should be learnt from integrin antibodies for the treatment of other diseases. For example, Tysabri (also called natalizumab), an antibody which blocks α_4_ integrins and inhbits the α_4_-mediated adhesion of leukocytes to their counterreceptor (s) (Minagar et al. 2000; O’Connor et al. 2004, 2005). Although the specific mechanism(s) by which tysabri exerts its effects in multiple sclerosis (MS) have not been fully characterized, Tysabri was initially approved by the Food and Drug Administration (FDA) in U.S.A. in November, 2004 for the treatment of patients with relapsing forms of MS, but was withdrawn by the manufacturer three months later after three patients developed progressive multifocal leukoencephalopathy (PML), a serious viral infection of the brain, in the drug’s clinical trials, FDA then put clinical trials of the drug on hold, allowing them to resume a year later after confirming that there were no additional cases of PML. In June 2006, the FDA resumed marketing of Tysabri with a restricted distribution program. Tysabri is indicated for use as monotherapy, because we do not know enough about how its use with other immune modifying drugs could impact risk. (www.fda.gov/cder/drug/infopage/natalizumab).

### Other antibodies

α_1_β_1_ and α_2_β_1_ integrins play a significant role in the VEGF-driven angiogenesis. Ha 31/8 and Ha 1/29 are antibodies against α_1_ and α_2_ integrin subunits which were reported to inhibit endothelial cells in a gradient of immobilized collagen I assay (haptotaxis) by <40%, whereas the combination of both antibodies synergized to reach <90% inhibition (Alghisi and Ruegg, 2006). Consistent with these results, administration of both the anti-*α*_1_ and the anti-*α*_2_ antibodies to nude mice bearing a human A431 squamous cell carcinoma xenograft suppressed angiogenesis by <60% and tumor growth by >40% (Senger et al. 2002). Interestingly, preclinical studies with monoclonal antibodies (MAbs) against lactadherin, a glycoprotein of the milk fat globule membrane was found that there was a clear increase in VEGF-like proangiogenic activity (Taylor et al. 1997; Silvestre et al. 2005) when lactadherin is added back exogenously to the ischemic muscles. An investigation has further identified lactadherin as a physiological ligand of α_V_β_3_ and α_V_β_5_, thus confirming a proangiogenic activity of these integrins in the VEGF-dependent neovascularization in adult mice, but not in embryos. Animal test showed that the expression of Flk-1 (VEGFR-2) is elevated in β_3_-deficient mice, indicating that α_V_β_3_ can control the amplitude of the VEGF response by controlling the Flk-1 level or activity (Reynolds et al. 2004). In vitro, anti-α_5_β_1_ function-blocking mAbs (NKI-SAM-1, JBS5, or IIA1) inhibited adhesion in a 72% to 100% range depending on the cell line used. This result was further confirmed in vivo in an angiogenesis assay treated with fibroblast growth factor 2 (Kim et al. 2000). The anti-α_5_β_1_ M200 antibody (Volociximab) is another chimeric monoclonal antibody of α_5_β_1_ integrin that blocks tumor growth and metastasis. M200 binds to α_5_β_1_ integrin on activated endothelial cells with high binding affinity and inhibits in vitro tube formation induced by VEGF and/or bFGF, suggesting a mechanism of action independent of growth factor stimulus. In fact, inhibition of α_5_β_1_ function by M200 induced apoptosis of actively proliferating, but not resting endothelial cells (Ramakrishnan et al. 2006).

## Disintegrins, RGD-Based Peptides and Small Molecule Integrin Antagonists

The “disintegrin” terminology was initially applied in 1990 to describe a family of cysteine-rich, RGD-containing proteins from viper venom toxins that inhibit platelet aggregation and integrin-mediated cell adhesion (Gould et al. 1990; Niewiarowski et al. 1994; McLane et al. 2004). Studies of RGD-containing proteins in venom toxins have been found that a number of them, such as contortrostatin, salmosin and bitistatin (Markland et al. 2001; Zhou et al. 1999; Swenson et al. 2004; Golubkov et al. 2003; Kang et al. 1999; Chung et al. 2003; McQuade et al. 2004), are able to inhibit tumor growth and angiogenesis. Echistatin has been found to induce a decrease of both auto-phosphorylation and kinase activity of pp125FAK, suggesting inhibitory activity in processes integral to angiogenesis, such as cell growth, survival, and migration (Della et al. 2000). Triflavin was found to interact with either α_IIb_β_3_ on platelet membranes, resulting in inhibition of platelet adhesion, secretion, and aggregation in injured arteries, or α_V_β_3_ on SMCs subsequently inhibiting cell migration and proliferation (Sheu et al. 2001). Triflavin also blocks neuronal sprouting and the induction of hyperalgesia induced by peripheral nerve injury (Fu et al. 2004). Recently, soluble RGD peptides have been demonstrated to induce apoptosis by inducing conformational changes in procaspases, leading to increased oligomerization and subsequent autoprocessing of these enzymes (Buckley et al. 1999). In addition, RGD-containing proteins from venom toxins (e.g. salmosin, contortrostatin, rhodostomin and accutin) were also found to induce apoptosis (Chung et al. 2003; Zhou et al. 1999; Wu et al. 2003; Yeh et al. 1998). It is still not clear whether these proteins’ apoptotic induction is through interaction with integrins or through a different apoptotic pathway since Jan et al. in a recent issue of Cell have shown that integrins may regulate apoptosis, through caspase-independent mechanisms (Jan et al. 2004). These data have shown the potential for these RGD-containing snake venom proteins to function as integrin antagonists as well as anti-angiogenic and antimetastatic compounds, leading to drug development for therapeutic usage (Markland et al. 2001; Kerr et al. 2002; Hallahan et al. 2001; Coller, 2001).

The integrins that bind to RGD peptides are generally over-expressed in angiogenic vessels. In certain cancer, the tumor cells also express RGD-binding integrins. A vast body of preclinical and clinical literature exists on the use of RGD-based integrin antagonists in cardiovascular disease and cancer (Tucker, 2003; McQuade and Knight, 2003; Kumar et al. 2003; Shimaoka and Springer, 2004). A cyclic pentapeptide called EMD66203 [cyclic **L**-Arg-**L**-Gly-**L**-Asp-**D**-Phe-**L**-Val (RGDfV) peptide or cyclo(-Arg-GlyAsp-D-Phe-Val)](Aumailley et al. 1991) was shown preferential inhibition of vitronectin binding to the α_V_β_3_ rather than to the α_IIb_β_3_ (Frieser et al. 1996). Further modification of the EMD66203 led to the synthesis of EMD121974, an RGD-containing pseudopeptide (c(RGDfV)) or cyclo(Arg-Gly-Asp-D-Phe-[NMe]Val) also known as cilengitide (Dechantsreiter et al. 1999). Structural study revealed that the D-amino acid in this peptide is found preferentially in position i + 1 of a β II’ turn, a characteristic for its biological activity. EMD121974 is also a dual α_V_β_3_/α_V_β_5_ integrin antagonist with interesting biochemical and biological features to be tested in cancer therapy (Belvisi et al. 2005, 2006). The crystal structure of the extracellular segment of integrin α_V_β_3_ in complex with EMD121974 revealed that the pentagonal peptide inserted into a crevice between the propeller and βA domains on the integrin head (Xiong et al. 2002). EMD 121974 was demonstrated to be an α_V_-integrin antagonist and a potent inhibitor of angiogenesis, by inducing apoptosis of growing endothelial cells through inhibition of their α_V_-integrin interaction with the matrix proteins vitronectin and tenascin (Taga et al. 2002).

ST1646, an RGD-containing pseudopeptide, is a potent, highly selective α_V_β_3_/α_V_β_5_ integrin antagonist, equipotent to or more potent than the well-characterized integrin antagonists c(RGDfV) (Belvisi et al. 2006; Haier et al. 2002). The structure docking model for the ST1646-α_V_β_3_ complex has confirmed that, similarly to the crystal structure of the EMD121974-α_V_β_3_ complex, the ligand seems to interact mainly through electrostatic forces in a rather shallow cleft and that essentially no hydrophobic interactions can be observed (Belvisi et al. 2005). In an in vitro anti-angiogenic activity assay, ST1646 inhibited HUVEC proliferation with potency similar to EMD121974 (IC_50_, 2.9 and 4.4 μmol/L for the two compounds, respectively). The inhibitory effect was reversible. In an in vivo antiangiogenic activity assay as determined by daily administration of ST1646 (30 μg/embryo) with CAM (chick chorioallantoic membrane) assay at day 9 via a gelatin sponge implant and at day 12 for histologic analysis, showed significant inhibition of the angiogenic response triggered by both FGF2 and VEGF (p < 0.001) (Belvisi et al. 2005).

SCH 221153, an RGD-based peptidomimetic, inhibits the binding of the disintegrin, echistatin to α_V_β_3_ and α_V_β_5_ with similar potency, according to IC_50_ values of 3.2 nM and 1.7 nM, respectively (Kumar et al. 2001). SCH 221153 inhibits FGF2 and VEGF-induced endothelial cell proliferation in vitro according to IC_50_ equal to 3–10 μM (Kumar et al. 2001). Monsanto-Searle (St. Louis, MO) has reported an orally compound SC-68448 which inhibited α_V_β_3_-mediated endothelial cell proliferation in a dose-dependent manner but did not inhibit tumor cell proliferation, suggesting that effects on endothelial cell proliferation were not due to SC-68448-induced cytotoxicity. SC68448 was 100-fold more potent as a functional inhibitor of α_V_β_3_ versus α_IIb_β_3_ (Carron et al. 2000). Integrin α_IIB_β_3_ expressed mainly on platelet membrane plays a crucial role in platelet aggregation and thrombus formation, and recently was reported to have a role in increasing the risk of metastases in renal cell carcinoma in men (Kallio et al. 2006). Haubner and his co-workers reported that ^18^F-Galacto-RGD is a highly α_V_β_3_-selective tracer for positron emission tomography (PET) (Haubner et al. 2004, 2001). Molecular imaging with ^18^F-Galacto-RGD and PET provides important information for planning and monitoring anti-angiogenic therapies targeting the α_V_β_3_ integrin (Beer et al. 2006). Meerovitch et al. demonstrated BCH-14661 and BCH-15046, RGD peptidomimetic compounds are as apoptotic inducers for endothelial cells by causing cell detachment-dependent when cells are grown on RGD-containing integrin ligand vitro-nectin and fibronectin. BCH-14661 was specific for integrin α_V_β_3_, whereas BCH-15046 nonselectively antagonized α_V_β_3_, α_V_β_5_, and α_5_β_1_ (Meerovitch et al. 2003). A 20 amino acid N-terminal peptide of angiocidin was reported to promote α_2_β_1_- dependent adhesion of K562 cells, disrupt human umbilical vein endothelial cell tube formation and inhibit tumour growth as well as angiogenesis in a mouse model (Sabherwal et al. 2006). Angiocidin has also been reported to inhibit angiogenesis through binding collagen and integrin α_2_β_1_ present on many tumour cells (Sabherwal et al. 2006).

The most selective nonpeptidic α_5_β_1_ antagonist SJ749 showed a reduced proliferation of astrocytoma cell lines dependent on α_5_β_1_ expression levels and cell culture conditions, underlining the importance of α_5_β_1_ as a target for anticancer therapies (Marinelli et al. 2005; Maglott et al. 2006). A non—peptide RGD mimetic, S36578-2, was also developed and demonstrated as highly selective antagonist of both α_V_β_3_ and α_V_β_5_ integrins that was able to induce detachment, caspase-8 activation, and apoptosis in human umbilical endothelial cells (HUVECs) plated on vitronectin (Maubant et al. 2006). Reinmuth and his co-works demonstrated that S-247, another α_V_β_3_/α_V_β_5_ integrin antagonist, showed significant antimetastatic and antiangiogenic activity and impaired both endothelial and hVSMC/pericyte function *in vitro* and *in vivo* (Reinmuth et al. 2003; Harms et al. 2004).

The integrin-induced signaling cascades have also been demonstrated to impact tumor cell survival, cell migration, and angiogenesis. It is known that transforming growth factor (TGF)-beta suppresses breast cancer formation by preventing cell cycle progression in mammary epithelial cells (MECs). During the course of mammary tumorigenesis, genetic and epigenetic changes negate the cytostatic actions of TGF-beta, thus enabling TGF-beta to promote the acquisition and development of metastatic phenotypes. TGF-β stimulation can induce α_V_β_3_ integrin expression in a manner that coincides with epithelial-mesenchymal transition (EMT) in MECs. Introduction of siRNA against β_3_ integrin can block TGF-β induction and also prevent TGF-β stimulation of EMT in MECs (Galliher and Schiemann, 2006). Therefore, antagonists of growth factor receptors (Cardones et al. 2006; Wick et al. 2006) can be used for anti-cancer therapy. Indeed, the recognition of potent, sequence-selective gene inhibition by siRNA oligonucleotides and rapid adoption as the tool of choice in cell culture has generated the expectation for their use to improve targeted therapeutics (Elbashir et al. 2002; Paddison et al. 2003; Carpenter and Sabatini, 2004; Ganju and Hall, 2004). The prospects of siRNA to be a therapeutic tool were enhanced by their double-stranded RNA (dsRNA) oligonucleotide nature, resembling antisense, ribozymes and gene therapy (Song et al. 2003; Davidson et al. 2004). Silencing integrin α_V_ expression by siRNA can inhibit proliferation and induce apoptosis in integrin α_V_ over-expressing MDA-MB-435 human breast cancer cells (Cao et al. 2006). Lipscomb and his co-workers demonstrated that siRNA oligonucleotides targeted to either subunit of the α_6_β_4_ integrin reduced cell surface expression of this integrin and resulted in decreased invasion of MDA-MB-231 breast carcinoma cells (Lipscomb et al. 2003). Recently gene transfer of antisense α_V_ and β_3_ expression vectors was demonstrated to downregulate α_V_ and β_3_ in HepG2 tumours established in nude mice, inhibit tumour vascularization and growth, and enhance tumour cell apoptosis, suggesting that anti-sense gene therapy targeting α_V_ integrins could be as an approach to treat hepatocellular carcinomas (Li et al. 2007).

A study using SUM-159 breast carcinoma cell line showed that decreased expression of the α_6_β_4_ integrin led to enhanced apoptosis. Recombinant VEGF is able to significantly inhibit the cell death observed in the β_4_-deficient cell line. The specificity of α_6_β_4_ in both in vitro and in vivo assays showed that reexpression of the β_4_ subunit into the β_4_-deficient cell line could rescue the functional phenotype (Lipscomb et al. 2005).

## Conclusions

In this review the potential roles of integrin in tumor progression and cancer were discussed. Evidence presented here indicates that integrins represent highly appropriate pharmacological targets as based upon the beneficial effect of integrin antibodies and antagonists in cancer treatment. A number of the integrin antibodies and antagonists are now in clinical trials, determining their effect on angiogenesis, metastasis and tumour growth (Table 2).

## Figures and Tables

**Table 1:** The integrin family of proteins and their ligands.

β_1_		
	α_1_	Colls, laminins
	α_2_	Colls, laminins, chondroadherin
	α_3_	Laminins (such as laminin-1, -5, -8, -10,
		and -11), Fn, thrombospondin, TIMP-2, uPAR, collagen, epiligrin, entactin
	α_4_	Fn, VCAM
	α_5_	Fn, Fg, uPAR
	α_6_	Laminins, merosin (laminin α2 chain), kalinin
	α_7_	Laminins, merosin (laminin α2 chain),
	α_8_	Fn, vitronectin, Tn-C, osteopontin, and nephronectin
	α_9_	angiostatin, Tn-C, osteopontin, and ADAMs, VCAM-1, tTG,
	α_10_	Colls
	α_11_	Colls
	α_V_	Fn, vitronectin
β_2_		
	α_L_	ICAM-1, -2 and -3
	α_M_	Fg, ICAMs, iC3b, factor-Xa, denatured ovalbumin
	α_X_	Fg, iC3b
	α_D_	VCAM, ICAMs
β_3_		
	α_IIb_	Coll, Fn, vitronectin, Fg, vWF, thrombospondin
	α_V_	Fn, vitronectin, Fg, vWf, thrombospondin, FGF-2, MMP-2 and some ADAM proteins
β_4_		
	α_6_	Laminins
β_5_		
	α_V_	Vitronectin, uPAR
β_6_		
	α_V_	Fn, Tn
β_7_		
	α_4_	Fn, VCAM, MAdCAM
	α_E_	E-cadherin
	α_V_	Colls, laminins, Fn
β_N_		
	α_V_	Fn, Colls
β_8_		
	α_V_	Vitronectin, Fn

The various heterodimeric combinations of α and β subunits. Abbreviations used are: Colls: collagens; Fn: fibronectin; TIMP-2: tissue inhibitor of metalloproteinase; uPAR: urokinase-type plasminogen activator (uPA) receptor; VCAM: vascular cell adhesion molecule; Fg: fibrinogen; Tn-c: tenacin-C; ADAMs: a disintegrin and metalloproteinase proteins; tTG: tissue-type transglutaminase; iC3b: inactivated complement component 3b; ICAM: intercellular cell adhesion molecule; vWf: von Willebrand factor; FGF-2: fibroblast growth factor 2; MMP: matrix metallo-proteinases and MAdCAM: mucosal addressin cell adhesion molecule.

**Table 2:** Integrin inhibitors in clinical development as anticancer agents.

Antibodies	Other names	Target integrin	Comments on highest phase reached	Company	References
LM609	Vitaxin, MEDI-552	α_V_β_3_	Currently in Phase II	Scripps Research Institute	Brooks et al. 1994; [Bibr b77-pmc-2008-057][Bibr b134-pmc-2008-057]
CNTO95		α_V_β_3_, α_V_β_5_	Currently in phase I	Centocor. Medarex	[Bibr b196-pmc-2008-057][Bibr b146-pmc-2008-057]
Ha31/8		α_1_β_1_			Senger et al. 2002
17E6		α_V_β_3_		Merck	Mitjans et al. 1995, 2000
Ha1/29		α_2_β_1_			Senger et al. 2002
NKI-SAM-1, JBS5		α_5_β_1_			Francis et al. 2002
M200	Volociximab Eos-200-4	α_5_β_1_	Currently in phase II	Protein design Labs	Protein Design Labs, www.pdl.com
**Peptides**					
SCH 221153					Kumar et al. 2001
EMD 121974	Cilengitide	α_V_β_5_	Currently in phase II	Merck KGaA, EMD Pharmaceuticals, National cancer Institute	Taga et al. 2002
ST1646		α_V_β_3_			Belvisi et al. 2005
angiocidin		α_2_β_1_			Sabherwal et al. 2006
^18^F-Galacto-RGD					Haubner et al. 2004, 2001
**Nonpeptidic**					
SJ749		α_5_β_1_			Marinelli et al. 2005; Maglott et al. 2006
E7820		α_2_ subunit	Phase I	Eisai Medical Research	
S36578-2		α_V_β_3_, α_V_β_5_			Maubant et al. 2006
S-247		α_V_β_3_, α_V_β_5_			Reinmuth et al. 2003; Harms et al. 2004
